# Alterations in Gut Microbial Co-Abundance Networks in Metabolic Syndrome: A Population-Based Cross-Sectional Study

**DOI:** 10.3390/microorganisms13122759

**Published:** 2025-12-04

**Authors:** Yiting Fang, Xi Meng, Rong Cao, Jianhang Li, Hui Cai, Peihua Liao, Xingfen Yang, Guiyuan Ji, Wei Wu

**Affiliations:** 1 NMPA Key Laboratory for Safety Evaluation of Cosmetics, Guangdong Provincial Key Laboratory of Tropical Disease Research, School of Public Health, Southern Medical University, Guangzhou 510515, China; fangyiting1207@163.com (Y.F.); mengxi.009@163.com (X.M.); 2 Guangdong Provincial Institute of Public Health, Guangdong Provincial Center for Disease Control and Prevention, Guangzhou 511400, China; caorongjsnt@126.com (R.C.); lijh325@mail2.sysu.edu.cn (J.L.); caihui1020@foxmail.com (H.C.); 3 Xinjiang Uighur Autonomous Region Center for Disease Control and Prevention, Urumqi 830002, China; 18999184912@163.com

**Keywords:** gut microbiota, metabolic syndrome, co-abundance, cross-sectional study

## Abstract

Metabolic syndrome (MetS) is a cluster of risk factors for cardiovascular diseases and type 2 diabetes. Gut microbiota dysbiosis has been implicated in the pathogenesis of MetS, but the mechanisms remain poorly understood. This study investigates gut microbiota interaction networks in MetS and explores their potential role in host metabolic regulation. In this population-based cross-sectional study, 221 MetS patients and 382 healthy controls were analyzed. Co-abundance network analysis was used to examine microbial interactions across the study. Significant differences in microbial co-abundance patterns were observed between MetS and healthy participants. In MetS, the gut microbiota displayed fewer and generally weaker co-abundance correlations compared with healthy controls. These changes appear to be more strongly associated with the synergistic effects of microbial interactions than solely with the abundance of individual taxa investigated here. Specific microbiota combinations were found to influence key metabolic functions, contributing to MetS development. The findings suggest that microbial interactions, rather than the abundance of individual bacteria, are associated with MetS. This study provides new insights into the role of disrupted gut microbiota networks in MetS pathogenesis.

## 1. Introduction

Metabolic syndrome (MetS) refers to a cluster of clinical conditions, including obesity, hyperglycemia, dyslipidemia and hypertension [1]. It is a major risk factor for both type 2 diabetes mellitus and cardiovascular diseases [2]. MetS affects approximately one-quarter of the global population [3]. The pathogenesis of MetS is multifactorial, involving both genetic predisposition and environmental influences. Increasing evidence suggests that gut microbiota dysbiosis is a critical factor influencing the development of MetS [4,5].

However, most existing studies focus on changes in microbial diversity, species composition, individual taxa, or metabolic pathways, while often overlooking the role of microbial interactions. In fact, the gut microbiota constitutes a complex ecosystem in which symbiotic and competitive relationships among bacterial species modulate community structure, thereby influencing host health [6].

In recent years, the exploration of microbial interactions through co-abundance network analysis has emerged as a prominent approach in disease research. By characterizing the symbiotic pattern of microbial communities, this method offers a novel perspective for elucidating disease mechanisms. It has been widely applied in studies of diverse conditions, including hypertension, inflammatory bowel disease, and obesity [7,8].

Within these ecological networks, certain taxa act as keystone species—microbes that exert a disproportionately large influence on community stability and function relative to their abundance [9,10]. Such species can shape the overall network architecture by regulating interactions among other community members and maintaining ecological balance [11]. Identifying these keystone taxa is, therefore, essential for understanding how alterations in microbial interactions may be associated with disease, as explored in the subsequent Empirical Presence–Abundance Interrelation (EPI) analysis and the identification of *Eubacterium* as a potential keystone genus.

**Figure 1:**
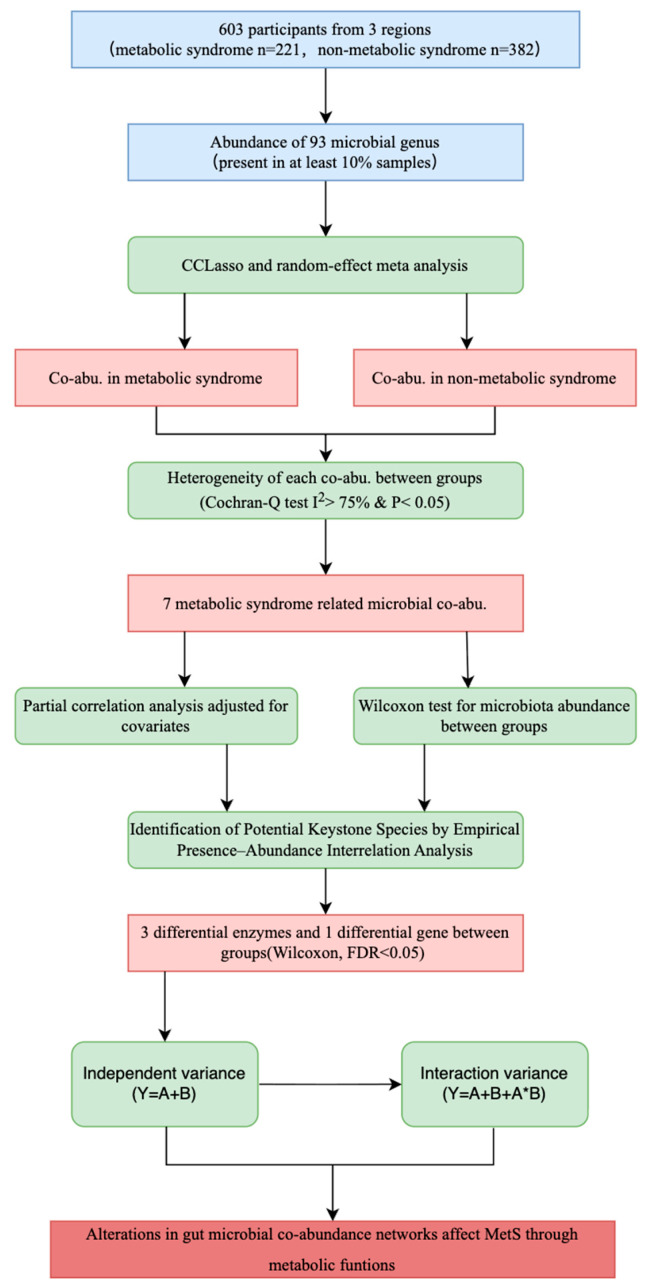
The analysis process of this study. A total of 221 patients with metabolic syndrome and 381 non-metabolic syndrome participants from three regions in a certain city of Xinjiang were included in this study. For the inference of microbial co-abundance networks, we incorporated at least 93 bacterial genera present in 10% of the samples.

Notably, evidence linking microbial interactions to MetS remains limited. This study was based on the study from the 2023 Chronic Disease and Risk Factor Surveillance Project conducted in a city in Xinjiang, China. The study encompassed three farms in the region and included 221 patients with MetS and 382 non-MetS. Using co-abundance network analysis, we systematically investigated the characteristics of intestinal microbial interactions in MetS, with a focus on keystone species that may affect microbial community structure (Figure 1). Our findings indicate significant alterations in microbial co-occurrence patterns among patients with MetS, providing new insights into the mechanisms underlying gut microbiota dysbiosis in this condition.

## 2. Materials and Methods

### 2.1. Study Design

A population-based cross-sectional study on chronic diseases and associated risk factors was conducted in a city in Xinjiang, China. The study was carried out in three farms under the jurisdiction of the city in 2023, enrolling 603 participants. Comprehensive data, including socio-demographic characteristics, disease status, and gut microbiota profiles, were collected. The participants were categorized into two groups based on the presence of MetS: the MetS group (n = 221) and the non-MetS group (n = 382). The baseline characteristics of these groups are summarized in Table 1. The MetS group had a mean age of 56.22 years (SD: 11.53) and comprised 59.73% males. The non-MetS group had a mean age of 50.81 years (SD: 13.30) with 37.43% males. The distribution of participants by smoking status, alcohol consumption, physical activity levels, and dietary intake for each group is detailed in Table 1.

### 2.2. Microbial Data Generation and Processing

All participants provided written informed consent prior to sample collection. Each participant received a sterile stool sampler, an ice pack, and detailed instructions for sample collection and storage. Immediately after collection, stool samples were placed in thermal containers with ice packs and delivered to the designated collection site within 24 h. Samples were transported to the laboratory and immediately stored at −80 °C in an ultra-low temperature freezer until analysis.

Fecal DNA was extracted using the FastPure Stool DNA Isolation Kit (MEIJIYUHUA, Shanghai, China) in accordance with the manufacturer’s protocol, with purity confirmed by A260/A280 ratios (≥1.8). The V1–V9 regions of bacterial 16S rRNA genes were amplified using primers 27F/1492R from the 16S Barcoding Kit (SQK-16S024, Oxford Nanopore Technologies, Oxford, UK). Purified PCR products were used for library preparation and sequenced on a MinION Mk1C platform (Oxford Nanopore Technologies, Oxford, UK). Raw data were base-called and demultiplexed using Guppy (v4.0.15). Taxonomic assignment was performed by aligning sequences to the Emu RDP database (v11.5) using Minimap2, with Kraken2 used supplementarily to validate classifications and improve resolution. Taxonomic assignment was performed by aligning sequences to the Emu RDP database (v11.5) [12] using Minimap2 (v2.24), with Kraken2 (v2.1.1) used supplementarily to validate classifications and improve resolution. Predicted metagenomic functions, including enzyme commission (EC) numbers, KEGG orthologs (KOs), and MetaCyc pathways, were inferred using PICRUSt2 (v2.3.0) based on 16S rRNA gene data.

### 2.3. The Definition of Metabolic Syndrome

Individuals were diagnosed with MetS according to the Chinese Guidelines for the Prevention and Treatment of Type 2 Diabetes Mellitus (2020) [13]. A diagnosis of MetS requires the presence of at least three of the following criteria:

- Abdominal obesity: waist circumference ≥ 90 cm for men and ≥85 cm for women.
- Hyperglycemia: fasting plasma glucose (FPG) ≥ 6.1 mmol/L and/or 2 h postprandial plasma glucose (2hPG) ≥7.8 mmol/L and/or diagnosed and treated for diabetes.
- Hypertension: blood pressure ≥ 130/85 MMHG and/or hypertension has been diagnosed and treated.
- Hypertriglyceridemia: fasting triglycerides (TG) ≥ 1.70 mmol/L.
- Low high-density lipoprotein cholesterol (HDL-C): HDL-C < 1.04 mmol/L.

### 2.4. Construction of Co-Abundance Network

In this study, the CCLasso method (component data correlation inference based on the Least Absolute Shrinkage and Selection Operator) was employed to construct the microbial interaction network [14,15]. Unlike traditional Pearson correlation, which assumes absolute microbial abundances, CCLasso accounts for the compositional nature of microbiome data, thereby reducing the risk of spurious correlations. By incorporating a least-squares framework with an L1 penalty, CCLasso infers correlations among latent variables while mitigating overfitting caused by collinearity or high dimensionality. Moreover, comparative analyses have demonstrated that CCLasso outperforms commonly used methods, such as SparCC, in accurately recovering network edges from compositional data. We constructed co-abundance networks for the MetS and non-MetS groups using the CCLasso method, based on the abundance data of 93 microbial genera with a sample prevalence ≥10% in the study. Analyses were performed independently for each of the three study regions. In total, six independent microbial co-abundance networks were constructed, representing the characteristic microbial interactions between MetS and non-MetS participants in the three study regions.

### 2.5. Meta-Analysis of Co-Abundance Networks in Different Regions

We then applied the metacor ( ) function from the R package *meta* (v.6.5.0) to integrate the microbial co-abundance networks from the three regions [16]. The function was implemented using the correlation coefficients (cor) of each microbial association derived from CCLasso and the corresponding sample sizes (n) for each region as input parameters. A random-effects model was employed to analyze the effect size of microbial co-abundance correlations, and the Benjamini–Hochberg method was applied to calculate the false discovery rate (FDR) to control false positives.

### 2.6. Heterogeneity Test of Co-Abundance Networks

In this study, a meta-analysis was performed using the metagen ( ) function [16] from the R package *meta* to compare the differences in microbial co-abundance between participants with and without MetS. This analysis was conducted by providing the pooled Fisher’s Z-transformed correlation coefficients and their standard errors from each group as inputs to the function. Heterogeneity between groups was assessed by calculating the effect size of co-abundance in each group, expressed as the pooled co-abundance coefficient in the meta-analysis. Co-abundance relationships with an I^2^ value greater than 75% and a *p*-value less than 0.05 in Cochran’s Q test were considered to indicate significant heterogeneity, according to pre-specified criteria [17].

### 2.7. Effect of Covariates on Co-Abundance Networks Associated with MetS

Age, sex, smoking status, alcohol consumption, physical activity, and dietary factors—including intake of grains, vegetables, fruits, dairy products, animal products, beans and nuts—were included as covariates in the analysis of microbial co-abundance associated with MetS (Table 1). The analytical procedure was as follows: First, Pearson correlation coefficients between all microbial genera involved in MetS-related co-abundance and each covariate were calculated to construct a correlation matrix. Subsequently, this matrix was adjusted for partial correlation using the cor2pcor ( ) function in the R package corpcor (v1.6.10) [18], which was applied to the full correlation matrix encompassing all microbial genera and covariates. This approach has been applied in prior microbiome network studies to control for confounding factors [7,8]. To evaluate the effect of adjustment, Cochran’s Q test was used to compare the difference in the co-abundance coefficient before and after adjustment, with *p* > 0.05 considered indicative of effective control of covariate influence.

### 2.8. EPI-Based Validation of Co-Abundance Results

To further validate the differential co-abundance results, we applied the EPI method, which identifies candidate keystone species from cross-sectional data by assessing their presence–impact on the abundance profiles of other taxa [19]. Three EPI definitions—D1 and D2 (distance-based measures of relative abundance distributions) and Q (a modularity-based measure from network science)—have been proposed. Application of these definitions in simulated cross-sectional data has been shown to successfully identify designated cornerstone species as clear keystone candidates.

### 2.9. Differences in Microbial Abundance

Abundance data for 13 microbial genera involved in differential co-abundance, 2011 EC, 5988 KOs, and 384 metabolic pathways (with a sample prevalence ≥10%) were processed using inverse rank transformation. In statistical analyses, we adjusted for five potential confounders—age, sex, smoking status, alcohol consumption, physical activity, and dietary factors by using linear regression models. To assess the differences between the MetS and the non-MetS group, we applied the Wilcoxon rank-sum test for between-group comparisons, with BH method used for multiple testing correction to control the FDR.

### 2.10. The Variance of Microbiologically Associated MetS-Related Functions Involved in Co-Abundance Was Estimated

A multistage analysis approach was used to assess microbial function variations associated with MetS [7]. For the identified MetS-associated microbial functions, binary linear regression models were applied to characterize the individual variation attributable to microbial genera involved in the co-abundance relationship:

(1) $$Y=\alpha +\beta_{1}\chi_{1}+\beta_{2}\chi_{2}+\varepsilon$$

Y represents the microbial pathway, EC, KO associated with MetS, α is the constant term, X_1_ and X_2_ are the independent variables, β_1_ and β_2_ are the regression coefficients, and ε denotes the error term.

Next, we introduced an interaction term between these two microorganisms into the model to evaluate its contribution to the explained variance of microbial metabolic pathways related to MetS: $Y=\alpha +\beta_{1}\chi_{1}+\beta_{2}\chi_{2}+\beta_{1*2}\chi_{1*2}+\varepsilon$. *p*-values were obtained using the F-test of the linear regression model, and the results were adjusted for multiple testing using the BH method.

## 3. Results

### 3.1. Microbial Interactions in Participants with and Without Metabolic Syndrome

Gut microbiota data from 221 participants with MetS and 382 participants without MetS were obtained from the 2023 Chronic Disease and Risk Factor Surveillance conducted in a city in Xinjiang, China. 93 bacterial genera present in at least 10% of participants (n = 603) were included in constructing gut microbial interaction networks (Figure 1).

**Figure 2:**
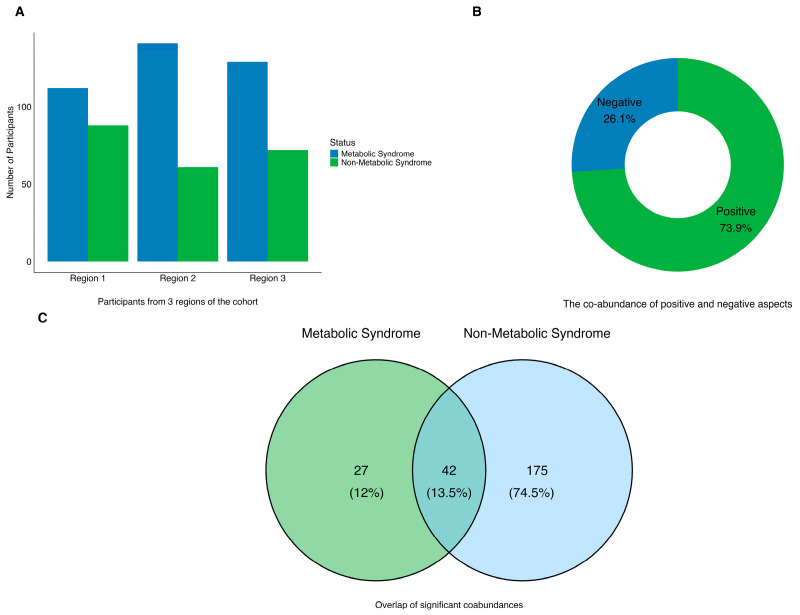

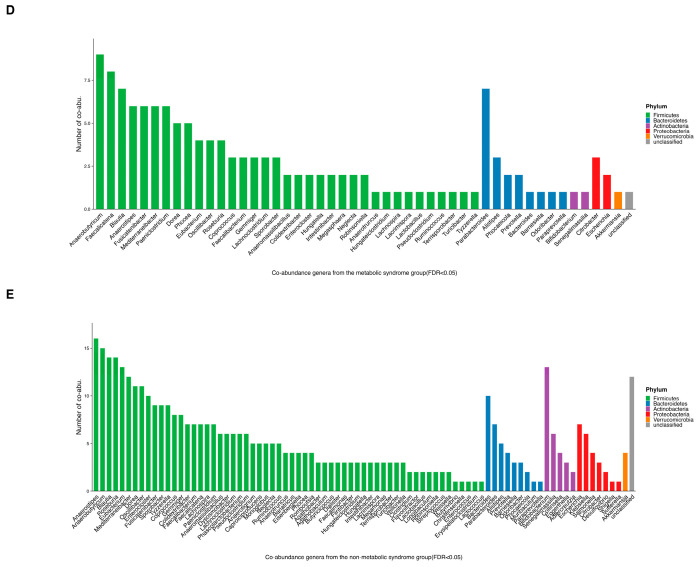
Microbial co-abundances in MetS and non-MetS. (**A**) Distribution of participants with MetS (blue) and non-MetS (green) across the three study regions (X-axis). (**B**) Summary of microbial interactions in MetS group based on co-abundance directions. (**C**) Overlap of significant microbial co-abundance (FDR < 0.05) between MetS and non-MetS. (**D**,**E**) Microbial genera with the most significant co-abundances (FDR < 0.05) in MetS (**D**) and non-MetS (**E**). For both panels, the bar height indicates the number of significant associations, and colors denote the phylum-level taxonomy. The phyla include: *Firmicutes*, *Bacteroidetes*, *Actinobacteria*, *Proteobacteria*, *Verrucomicrobia*, or unclassified.

The CCLasso method was employed to infer the co-abundance networks for each of the three regions separately (Figure 2A). Based on the co-abundance effect size, a random-effects meta-analysis was performed on the microbial co-abundance networks of the three regions. This analysis identified 69 co-abundance networks (FDR < 0.05) in participants with MetS. A substantially lower number of co-abundance networks was identified in participants with MetS (69 networks, FDR < 0.05) compared to those without MetS (217 networks, FDR < 0.05) (Figure 2C, Tables S1 and S2). The co-abundance networks in both groups were predominantly composed of five phyla.

The proportion of genera belonging to *Firmicutes* was similar in both groups (71.4% in MetS and 72.3% in non-MetS). However, the number of co-abundance genera ranged from 1 to 9 in MetS participants, compared with 1 to 16 in non-MetS participants (Figure 2D,E). Additionally, we determined the number of co-abundance directions in participants with MetS. Positive co-abundance (where one genus positively affects another) accounted for 73.9% of the entire network, which was higher than the 52% observed in participants without MetS (Figure 2B).

### 3.2. The Co-Abundance of Multiple Microorganisms Was Different Between Mets and Non-Mets

**Figure 3:**
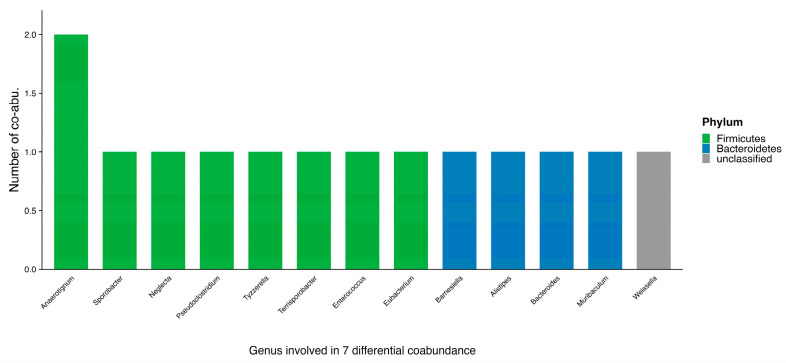
Microbial genera with differential co-abundance in MetS. The bar plot displays microbial genera (X-axis) against their number of differential co-abundances in MetS participants (Y-axis). Colors represent the phylum-level taxonomic classification.

The co-abundance of multiple microorganisms differed between participants with MetS and those without. A heterogeneity test was applied to assess whether the strength of each co-abundance differed between the two groups. The results revealed 7 co-abundances with significant differences in intensity (Cochran’s Q test, I^2^ > 75% and *p* < 0.05), involving 13 genera (Table 2, Figure 3). Analysis showed that only one of the seven co-abundances, *Sporobacter-Alistipes*, was weakened in non-MetS participants compared to MetS participants, while the remaining six co-abundances increased in intensity. Furthermore, the co-abundance between *Terrisporobacter* and *Eubacterium* exhibited differences in the direction of interaction, rather than merely a change in strength.

Partial correlation analysis [8] was then applied to assess potential bias from other influencing factors, including age, sex, smoking status, alcohol consumption, physical activity level and dietary factors, to verify the reliability of the observed co-abundance patterns. Comparison of partial correlation results before and after adjustment revealed no significant differences in microbial co-abundance patterns associated with MetS (Cochran’s Q test, *p* > 0.05), indicating that the identified associations were not influenced by confounding factors (Table S3).

We employed the EPI method to further validate the potential key species identified from the differential co-abundance analysis. Species included in this analysis were those with prevalence rates between 25% and 75% across all samples. This method evaluates the influence of each species’ presence on the abundance profiles of other species using three indicators: D1 and D2 and Q. Among the 41 species analyzed, most were not identified as key species by any of the three indicators (IsKeystone = FALSE). However, *Eubacterium* was identified as a keystone species by both the D1 and D2 indicators (IsKeystone (D1) = TRUE; IsKeystone (D2) = TRUE), while *Phascolarctobacterium* was identified only by D1 (IsKeystone (D1) = TRUE) (Table S4).

A group comparison analysis of abundance levels for the 13 genera involved in MetS-associated co-abundance was subsequently performed. The results indicated that only two genera, *Enterococcus* and *Alistipes*, exhibited significant differences between the two groups (Wilcoxon test, FDR < 0.05), suggesting that the MetS-associated co-abundance pattern partially corresponds to differences in microbial abundance (Table S5).

### 3.3. Microbial Interactions May Affect Metabolic Syndrome Through EC and KO

Microbial interactions may influence MetS through various metabolic functions, including enzymes ECs and KOs. Among the metabolic functions stable in ≥10% of the samples, no significant differences were observed in metabolic pathways between the MetS and control groups (Wilcoxon rank-sum test, FDR < 0.05). However, several enzyme- and gene-level features differed between groups: three enzymes (EC 2.5.1.46, EC 1.14.14.1, and EC 5.5.1.25) and one KO gene (K00809) were more abundant in the non-MetS group (Tables S6 and S7). These results suggest that functional differences between groups are limited to specific enzymatic activities rather than broad metabolic pathway alterations.

We hypothesized that the microbial interaction network may influence the onset and progression of MetS by regulating host metabolic functions. To test this, microbial interaction terms were incorporated into linear regression models to quantify the variance in metabolic functions explained by specific co-abundance relationships. By integrating microbial co-abundance interaction networks with metabolic function analyses, we elucidated potential regulatory mechanisms of bacterial cooperation in host metabolism. We quantified the additional explained variance (extra_var) for three significantly correlated microbial co-abundances across three enzymes and one KO gene, with all results adjusted for multiple testing (FDR < 0.05) (Table 3). Among these, the *Barnesiella–Anaerotignum* co-abundance contributed most strongly to the enzyme 2.5.1.46 (deoxyhypusine synthase) (extra_var = 0.0079, FDR = 2.4 × 10^−5^). The *Neglecta–Anaerotignum* pair also showed a strong association with the same enzyme (extra_var = 0.0088, FDR = 1.9 × 10^−5^), accounting for 18.4% of the total explained variance (*var*-int = 0.0479). In contrast, *Sporobacter–Alistipes* exhibited the highest relative contribution to the enzyme 1.14.14.1 (unspecific monooxygenase), although its absolute extra_var was smaller (0.0011, FDR = 6.7 × 10^−4^). Collectively, these findings suggest that specific microbial interactions, particularly those involving *Anaerotignum* with *Barnesiella* or *Neglecta*, may modulate host metabolic processes through enzyme-level functional modules associated with amino acid and cofactor metabolism.

## 4. Discussion

This study is the first to identify significant differences in gut microbiota interaction patterns between individuals with MetS and those without in northwest China. The MetS group exhibited a simpler co-abundance network (69 vs. 217 interactions) and a higher proportion of positive interactions (73.9% vs. 52%), and a narrower range of genus connections between genera (1–9 vs. 1–16), suggesting that metabolic abnormalities may be associated with a simplification and enhancement of the intestinal microbiome ecosystem [20,21].

The more complex interaction network in the non-MetS group may reflect the functional redundancy and stability of the healthy microbiota, whereas the increased positive interactions in the MetS group could indicate compensatory adaptation mechanisms to cope with microenvironmental changes linked to metabolic disorders [22,23]. Although both groups were dominated by *Firmicutes*, differences in structural characteristics and interaction directionality differences in the MetS group appear to represent a unique ecological imbalance, offering new insights into the intestinal microbiota patterns associated with MetS.

Seven microbial co-abundance relationships were significantly different between MetS and non-MetS populations, primarily in terms of changes in the strength and direction of interaction. In the non-MetS group, the co-abundance of six co-abundances (e.g., *Barnesiella-Anaerotignum*, *Neglecta-Anaerotignum*) exhibited stronger interaction strength. This enhancement may reflect the fine regulatory network maintained by gut microbiota in a healthy state, potentially supporting metabolic homeostasis through stable synergistic relationships among specific bacterial genera [8]. For example, the enhanced interaction between *Barnesiella* [24] and *Anaerotignum* [25] may be linked to the compensatory regulation of bile acid metabolism and Short-chain fatty acids (SCFAs) production. Reduced *Barnesiella* abundance observed in type 2 diabetes and high-fat diet–induced metabolic models suggests its potential protective role in maintaining glucose and lipid homeostasis, possibly through bile acid–mediated regulation of the TGR5–GLP-1 signaling pathway [26,27]. The genus *Anaerotignum* exhibits strong fermentative capacity. Genomic and metabolic modeling studies published in 2022 demonstrated that this bacterium can metabolize ethanol, lactic acid, various sugars, and amino acids to produce SCFAs, primarily propionate and acetate, via the propionate–acrylate pathway [28]. Clinical studies have shown that consuming fruit granola for breakfast increases *Neglecta* abundance, which helps reduce the risk of cardiovascular diseases [29]. The enhanced *Neglecta-Anaerotignum* interaction in the non-MetS population may represent a metabolic axis of ‘dietary fiber degradation-butyrate production’, potentially reducing cardiovascular disease risk by maintaining intestinal barrier integrity and regulating lipid metabolism [30,31]. Notably, *Terrisporobacter-Eubacterium* interaction exhibited a positive correlation in the MetS group and a negative one in the non-MetS group. This reversal in direction may reflect adaptive changes in microbiota ecology under metabolic disorders. In healthy individuals, *Eubacterium* [32], a key bacteria for butyrate production, likely inhibit the growth of inflammation-associated *Terrisporobacter* [33] through competitive mechanisms, forming a mutually restrictive equilibrium. However, in individuals with MetS, this healthy restrictive relationship appears to be disrupted, transforming into an abnormal coexistence pattern that is indicative of intestinal microbiota dysregulation. Studies have shown that a fiber-rich diet increases *Eubacterium* abundance [34], suggesting the potential of nutritional intervention to help restore healthy microbiota interaction patterns.

Although seven pairs of co-abundance relationships differed significantly between the MetS and non-MetS populations, the underlying biological mechanisms remain to be elucidated. The observed correlations likely reflect metabolic cooperation or competition among microbial genera that influence key host metabolic pathways. For instance, cooperative interactions between fiber-degrading and SCFA-producing bacteria (e.g., *Barnesiella-Anaerotignum*, *Neglecta-Anaerotignum*) may enhance butyrate and propionate synthesis, thereby improving intestinal barrier integrity, reducing endotoxin translocation, and modulating insulin sensitivity [25,26,27]. In contrast, disrupted or reversed interactions—such as *Eubacterium*-*Terrisporobacter*—may lead to impaired SCFA balance, increased production of inflammatory metabolites, or altered bile acid transformation [32,33,35]. Collectively, these network alterations could influence host lipid and glucose metabolism, inflammatory tone, and bile acid signaling, offering a potential ecological basis for the metabolic dysregulation observed in MetS.

Consistent with these network-level findings, the EPI analysis further identified *Eubacterium* as a key species, exerting significant influence on the abundance distribution of other taxa across multiple indicators (D1 and D2). This dual identification—through both differential co-abundance and EPI analyses—underscores *Eubacterium*’s central ecological position in maintaining microbial balance. Functionally, *Eubacterium* may act as a regulatory hub within the gut ecosystem, stabilizing metabolic processes through butyrate production and cross-feeding interactions. The disrupted *Terrisporobacter–Eubacterium* association observed in the MetS group may, therefore, be related to reduced network stability and metabolic resilience. Together, these findings highlight *Eubacterium*’s potential association with microbial network integrity and host metabolic health, meriting further validation through fecal microbiota transplantation or other controlled intervention studies.

In the non-MetS population, the co-abundance relationships between *Barnesiella–Anaerotignum* and *Neglecta–Anaerotignum* were notably enhanced, accompanied by higher additional variance contributions to metabolic functions such as EC:1.14.14.1 and EC:2.5.1.46. Unspecific monooxygenases are involved in oxidation–reduction reactions that contribute to detoxification and lipid oxidation, processes essential for maintaining metabolic homeostasis [36]. Deoxyhypusine synthase participates in polyamine biosynthesis, a pathway linked to cell proliferation and insulin signaling regulation [37]. The enhanced synergistic interactions between these genera may, therefore, support balanced redox metabolism and amino acid utilization, which could help maintain metabolic stability. These findings suggest that cooperative microbial relationships in healthy individuals may support functional resilience in key metabolic functions, potentially mitigating the risk of progression toward MetS.

Taken together, these findings provide a network-level ecological perspective on the gut microbiome in MetS and may help generate hypotheses regarding potential microbial biomarkers and regulatory targets. However, this study has several limitations. First, it should be noted that the functional profiles generated by PICRUSt2 are predictive and based on 16S rRNA gene compositions. While this approach is practical, its predictive accuracy is limited, particularly for non-model or poorly characterized microbiota. Therefore, future studies should integrate metagenomic or metabolomic analyses to validate and extend these functional predictions. Second, the imbalance in sample sizes between groups and potential confounding factors (e.g., differences in probiotics and antibiotics) may have influenced the statistical power and stability of the inferred microbial networks. Future studies should aim to minimize these biases through stricter participant matching, longitudinal designs, or adjustment for medication to better clarify microbiota–MetS associations. Third, the current analysis is primarily based on associations, and the biological hypotheses require further validation for causality through subsequent experiments, such as FMT, metabolomics, or in vitro models. Finally, the study population was predominantly Han Chinese and geographically restricted to three farms in Xinjiang, China, which may limit the generalizability of the findings to other ethnic and geographic populations. Future studies with larger and more diverse cohorts can help reduce confounding bias and improve the representativeness of the results. Additionally, multi-group studies and animal model experiments could further explore the functional mechanisms of the identified co-abundance modules in MetS development, providing a more reliable basis for potential intervention strategies.

## 5. Conclusions

This study reveals a significant association between disrupted gut microbiota interaction networks and MetS. In patients with MetS, the microbial co-abundance pattern is notably altered, characterized by reduced interactions and enhanced positive regulation. These changes are not driven by a single bacterial group’s abundance but result from the collective regulation of synergistic microbial interactions. This work provides an ecological systems perspective that complements traditional microbiome analyses, offering a new framework for understanding MetS pathogenesis and guiding microbiota-targeted interventions.

## Figures and Tables

**Table 1:** Summary of phenotypes between the metabolic syndrome and non-metabolic syndrome groups.

Phenotypes	Metabolic Syndrome (n = 221)	Non-Metabolic Syndromes (n = 382)
Age (sd)	56.22 (11.53)	50.81 (13.30)
Male (n, %)	132 (59.73)	143 (37.43)
Smoking (n, %)		
never	125 (56.56)	281 (73.56)
former	39 (17.65)	30 (7.85)
seldom	5 (2.26)	6 (1.57)
everyday	52 (23.53)	65 (17.02)
Alcohol consumption (n, %)		
never	129 (58.37)	229 (59.95)
<3 days/week	72 (32.58)	133 (34.82)
3–6 days/week	5 (2.26)	10 (2.62)
everyday	15 (6.79)	10 (2.62)
Physical activity (n, %)		
low	48 (21.72)	72 (18.85)
medium	80 (36.20)	112 (29.32)
high	93 (42.08)	198 (51.83)
Grains (sd)	357.70 (210.50)	369.19 (248.59)
Vegetables (sd)	476.64 (282.53)	466.31 (279.95)
Fruits (sd)	114.62 (153.02)	147.54 (207.15)
Dairy products (sd)	117.73 (184.43)	110.92 (126.43)
Animal products (sd)	177.85 (157.82)	202.98 (227.93)
Beans and nuts (sd)	39.98 (51.83)	43.17 (60.00)

**Table 2:** Co-abundance of seven microorganisms showing significant differences in metabolic syndrome. (Cochran’s Q test, I^2^ > 75%, *p* < 0.05).

Genuspair	TE_non-MetS	TE_MetS	seTE_non-MetS	seTE_MetS	Hetero_p	I^2^
*Sporobacter-Alistipes*	−0.1516305	−0.4312161	0.06792643	0.11578319	0.03727255	0.76947448
*Barnesiella-Anaerotignum*	0.23093215	0.02179268	0.05177804	0.06868028	0.01503502	0.83086279
*Neglecta-Anaerotignum*	0.32789629	0.03903557	0.08833262	0.06868028	0.00983372	0.84995738
*Pseudoclostridium-Bacteroides*	−0.2899035	−0.0958833	0.05177804	0.06868028	0.02408619	0.80347517
*Tyzzerella-Enterococcus*	0.30805484	0.05858727	0.05177804	0.06868028	0.00372682	0.88112707
*Terrisporobacter-Eubacterium*	−0.1575766	0.06119389	0.05177804	0.06868028	0.01097453	0.84542702
*Weissella-Muribaculum*	0.20824215	0.02352035	0.05177804	0.06868028	0.03174194	0.78319221

**Table 3:** Metabolic function variance estimated by the genus involved in co-abundances with and without interaction term among MetS and non-MetS.

Genuspair	Function	Description	Var_ind	Var_int	Extra_var	P_ind	FDR
*Sporobacter-Alistipes*	EC:1.14.14.1	Unspecific monooxygenase	0.02762911	0.02874302	0.00111391	0.00022366	0.00067097
*Barnesiella-Anaerotignum*	EC:2.5.1.46	Deoxyhypusine synthase	0.03834184	0.0462416	0.00789976	8.0578 × 10^−6^	2.4173 × 10^−5^
*Barnesiella-Anaerotignum*	EC:1.14.14.1	Unspecific monooxygenase	0.01542343	0.03128079	0.01585737	0.00943734	0.01415601
*Neglecta-Anaerotignum*	EC:2.5.1.46	Deoxyhypusine synthase	0.0390983	0.04792689	0.00882859	6.3634 × 10^−6^	1.909 × 10^−5^
*Neglecta-Anaerotignum*	EC:1.14.14.1	Unspecific monooxygenase	0.01465138	0.01794392	0.00329254	0.01193917	0.01790876
*Barnesiella-Anaerotignum*	KO:K00809	deoxyhypusine synthase [EC:2.5.1.46]	0.03850055	0.04644554	0.00794499	7.6685 × 10^−6^	7.6685 × 10^−6^
*Neglecta-Anaerotignum*	KO:K00809	deoxyhypusine synthase [EC:2.5.1.46]	0.03925636	0.04811791	0.00886155	6.057 × 10^−6^	6.057 × 10^−6^

## Data Availability

The original contributions presented in this study are included in the article and Supplementary Material. Further inquiries can be directed to the corresponding authors.

## Supplementary Materials

The following supporting information can be downloaded at: https://www.mdpi.com/article/10.3390/microorganisms13122759/s1, Table S1: Microbial co-abundance in metabolic syndrome. Using the CCLasso method and random meta-analysis, we identified 69 significant microbial co-abundancies (FDR < 0.05 in the random meta-analysis) among 94 bacterial genera in 221 patients with metabolic syndrome; Table S2: Microbial co-abundance in non-metabolic syndromes. Using the CCLasso method and random meta-analysis, we identified 217 significant microbial co-abundnesses in 94 genera among 382 participants without metabolic syndrome (FDR < 0.05 in the random meta-analysis); Table S3: The results of the partial correlation analysis of the co-abundance of 7 microorganisms before and after correction (Cochran’s Q test, *p* > 0.05); Table S4: Potential Keystone Species Identified via the Empirical Presence–Abundance Interrelation Method; Table S5: Results of comparison between groups for the abundance of genera associated with co-abundance associated with metabolic syndrome (wilcox-test, FDR < 0.05); Table S6: The difference in EC abundance between Metabolic syndrome and non-metabolic syndrome (wilcox-test, FDR < 0.05); Table S7: The difference in KO abundance between Metabolic syndrome and non-metabolic syndrome (wilcox-test, FDR < 0.05).

## Abbreviations

MetS	Metabolic syndrome
SD	Standard Deviation
EPI	Empirical Presence–Abundance Interrelation
EC	Enzyme commission
Kos	KEGG orthologs
FPG	Fasting plasma glucose
2hPG	2 h postprandial plasma glucose
TG	Triglycerides
HDL-C	High-density lipoprotein cholesterol
FDR	False discovery rate
SCFAs	Short-chain fatty acids
